# Protocol for evaluating iodine uptake and diffusion in sheep and pig meniscus tissue using contrast-enhanced micro-CT and UV-vis spectroscopy

**DOI:** 10.1016/j.xpro.2025.103951

**Published:** 2025-07-12

**Authors:** Federica Orellana, Annapaola Parrilli

**Affiliations:** 1 Empa - Swiss Federal Laboratories for Materials Science and Technology, 8600 Dübendorf, Switzerland

**Keywords:** Health Sciences, Microscopy, Model Organisms

## Abstract

Here, we present a protocol for evaluating iodine uptake and diffusion in sheep and pig meniscus tissue using contrast-enhanced micro-computed tomography (CT) and ultraviolet-visible (UV-vis) spectroscopy. We describe steps for sample fixation, a staining procedure with iodine-based contrast agents, and imaging using micro-CT. We then detail procedures for volumetric and radiodensity analyses, UV-vis spectroscopy, and data processing.

For complete details on the use and execution of this protocol, please refer to Orellana et al.^1^

## Highlights

• Assess iodine uptake and radiodensity via iodine-based staining and micro-CT imaging
• Guidance for sequential micro-CT scans over 24 days to track iodine uptake dynamics
• Integration of radiodensity analysis and UV-vis spectroscopy to quantify iodine uptake
• Steps for Avizo and MATLAB for segmentation, volumetric analysis, and data visualization

## Abstract (editor highlights)

Publisher’s note: Undertaking any experimental protocol requires adherence to local institutional guidelines for laboratory safety and ethics.

## Graphical abstract

**Figure:**
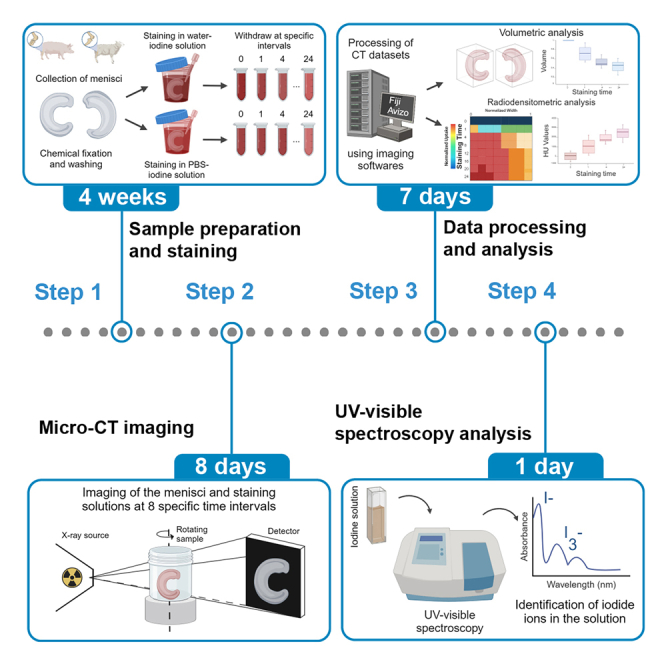

## Before you begin

Here, we describe the procedures used to evaluate iodine uptake and diffusion in sheep and pig meniscal tissues using contrast-enhanced micro-CT and UV-Vis spectroscopy, as applied in our associated research study.^1^ This protocol assesses iodine distribution in soft tissues over time using sequential micro-CT imaging after iodine staining. Although this protocol focuses on meniscal tissue from sheep and pigs, similar approaches can be used for other soft tissues or animal models that require diffusion-based contrast analysis.

### Institutional permissions

Review and/or approval by an ethics committee was not required for this study because the hindlimbs were collected from research animals after they were sacrificed at the study specific endpoints of other research projects.

Verify whether ethical approval is necessary for your research and ensure compliance with relevant regulations and guidelines for animal research.

**Table 1:** Micro-CT parameters

Sample	MICRO-CT analysis settings	Reconstruction parameters
Low resolution (25 or 30 μm)	Rotation step 0.25^°^ No. average frames 3 Frame rate 5 Voltage 70 kV, Current 70 μA Acquisition time 14 min	Filtered back-projection algorithm Small ring artifact reduction 75% Sinus window function
Mid resolution (8.5 μm)	Rotation step 0.3° No. average frames 5 Frame rate 1.5 Voltage 70 kV, Current 70 μA Acquisition time 64 min	Filtered back-projection algorithm Small ring artifact reduction 75% Sinus window function
High resolution (2.5 μm)	Rotation step 0.18° No. average frames 5 Frame rate 1 Voltage 90 kV, Current 60 μA Acquisition time 168 min	Filtered back-projection algorithm Small ring artifact reduction 75% Sinus window function

### Preparation of reagents

**Timing: variable**

1. Prepare the Formaldehyde fixative.
  a. Prepare a 4% formaldehyde solution in Phosphate-buffered saline (PBS, pH 6.9) can be prepared freshly from paraformaldehyde or used as a commercially available buffered formaldehyde solution (see key resources table).
  b. Store the fixative at 4°C for up to one month and prepare fresh if any precipitation or yellow discoloration occurs.

**CRITICAL:** Ensure that the fixative solution is handled under a fume hood with proper personal protective equipment to prevent exposure to hazardous chemicals.

***Note:*** This protocol was developed using formaldehyde as fixative. Other fixatives can be used; however, they could affect iodine binding and diffusion, and should be validated accordingly.

2. Prepare the PBS solution.
  a. Dissolve 1 tablet of PBS (see the key resources table) in 200 mL of distilled water. The resulting solution will have a pH of approximately 7.2–7.6.
  b. Store at 4°C for up to 1 month.

**CRITICAL:** Ensure the solution remains clear and free of contamination.

3. Prepare the Water-based Lugol’s Iodine Solution.
  a. Prepare an aqueous 1.25% w/v of I_2_ and 2.5% w/v of KI solution using a stirring instrument.
  b. Store in a light-protected container at 4°C for up to 1 month.

**CRITICAL:** Handle iodine and potassium iodide powders inside a fume hood to prevent inhalation. Avoid contact with metal surfaces to minimize corrosion risk; use non-metallic tools and containers when possible.

***Note:*** If stored, stir the solution again for a few minutes before use to resuspend any settled iodine.

***Note:*** Iodine-based contrast agents are the focus of this protocol. While other contrast agents can be used, their diffusion dynamics may differ, requiring optimization of concentration and staining duration.

4. Prepare the PBS-based Lugol’s Iodine Solution.
  a. Prepare a PBS-based 1.25% w/v of I_2_ and 2.5% w/v of KI solution.
  b. Store in a light-protected container at 4°C for up to 1 month.

**CRITICAL:** Handle iodine and potassium iodide powders inside a fume hood to prevent inhalation. Avoid contact with metal surfaces to minimize corrosion risk; use non-metallic tools and containers when possible.

***Note:*** If stored, stir the solution again for a few minutes before use to resuspend any settled iodine.

### Sample collection

**Timing: 1 h**

**Figure 1:**
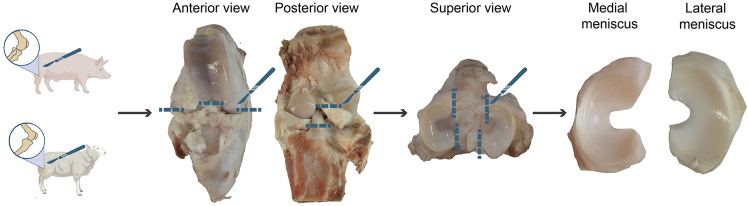
Sample preparation Sheep and pig stifle joints were dissected at the level of the collateral and cruciate ligaments to isolate the tibia with the menisci attached. The menisci were then carefully removed from the tibial plateau by cutting at the anterior and posterior horns.

5. Collect sheep and pig menisci.
  a. Use using the following materials:
    i. A saw, if bone cutting is required.
    ii. Scalpels and scissors to excise the meniscus.
    iii. Plastic containers of appropriate size for soaking the menisci in the fixative and staining solutions.
  b. Excise the knee with a cut approximately 10 cm above and below the tibiofemoral joint.
  c. Carefully dissected the menisci from the joint capsule (Figure 1).

**CRITICAL:** Exercise caution when using cutting tools. Wear anti-cut gloves if necessary.

## Key resources table

**Table:** 

REAGENT or RESOURCE	SOURCE	IDENTIFIER
**Biological samples**		

Meniscal samples from adult sheep (*Ovis aries*) and pig (*Sus domestica*)	MSRU, University of Zurich	N/A

**Chemicals, peptides, and recombinant proteins**		

Formaldehyde solution 4%, buffered, pH 6.9	Sigma-Aldrich	100496
PBS (phosphate-buffered saline)	Sigma-Aldrich	P4417
I_2_ (iodine)	Sigma-Aldrich	207772
KI (potassium iodide)	Sigma-Aldrich	221945

**Software and algorithms**		

X-Act v. 1.1, revision 22.11.1 2023-01-06	Rx Solutions	X-Act - Powerful and intuitive X-ray tomography software
Fiji v.1.54f	Schindelin et al.^2^	Fiji
Avizo v.2021.2	Thermo Fisher Scientific	Avizo Software | Materials Characterization Software | Thermo Fisher Scientific - CH
MATLAB R2019a	MathWorks	Get MATLAB

**Other**		

Micro-CT scanner	Rx Solutions	EasyTom XL - Flexible x-ray micro & ultra tomography system
Parafilm M sealing film	Merck	HS234526B
Spectrometer	Varian Cary 50 UV-vis	labstuff.eu - Varian Cary 50 Scan UV-VIS Photometer New
FiveEasy pH meter	METTLER TOLEDO	FiveEasy pH Meter I Compact & Economical

## Materials and equipment

### Micro-CT settings

***Note:*** Micro-CT scanning was performed at the Center from X-ray Analytics, Empa using Rx Solutions micro-CT scanner. Key scanning parameters, including X-ray voltage (kV), current (μA), resolution, rotation step, frame rate, number of average frames, were pre-optimized to ensure sufficient X-ray transmission through the samples and adjusted based on the specific characteristics of each sample. A comprehensive list of scanning parameters for the analyzed samples is provided in Table 1.

***Alternatives:*** There are other micro-CT systems can be selected such as Skyscan from Bruker and Xradia from ZEISS.

**CRITICAL:** In our case, personal protective measures were not required since we are using a closed micro-CT system, which is designed with built-in radiation safeguards. However, consult with the institutional safety board or radiation safety officer to ensure compliance with local safety protocols and verify any specific recommendations.

### Avizo software

***Note:*** Avizo, developed by Thermo Scientific, is a powerful 3D visualization and image analysis software used in several fields. It offers robust segmentation, image processing tools, and allows users to create high-quality images and animations.

***Alternatives:*** Other software alternatives for 3D CT data segmentation, analysis, and visualization include Dragonfly, VG Studio.

## Step-by-step method details

### Sample preparation and staining

**Timing: 4 weeks total (3 days for sample preparation; 24 days for staining)**

This section describes the fixation and iodine staining of meniscal tissues to preserve tissue structure and enable iodine diffusion. Sequential aliquot collection allows monitoring and quantification of iodine uptake dynamics over the 24-day staining period using imaging and spectroscopy.

**Figure 2:**
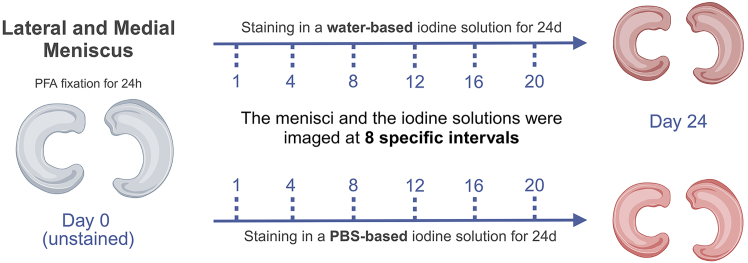
Sample preparation and staining Menisci were chemically fixed in formalin and stained in either water-based or PBS-based iodine solution for 24 days. Imaging of both the menisci and the iodine solutions was performed at 8 specific time points throughout the staining period.

1. Fix meniscal samples in 4% formaldehyde for 24 h.
2. Wash 3× with either water or PBS (approximately 100 mL for each wash) according to the subsequent staining solution.
3. Immerse menisci in either water-based (n = 6 sheep menisci, n = 6 pig menisci) or PBS-based (n = 6 pig menisci) Lugol’s iodine solution (1.25% I_2_, 2.5% KI) for up to 24 days (Figure 2). Staining was performed at approximately 22°C–25°C under static conditions.
  a. Use approximately 100 mL of staining solution per sample.
4. Collect 1 mL aliquots of the staining solutions at designated time points (0, 1, 4, 8, 12, 16, 20, and 24 days) into Eppendorf tubes. These time points are selected to capture the mid- and long-term dynamic process of iodine staining diffusion within the meniscus tissue. They can also serve as a reference for assessing diffusion in other similar tissues of comparable size.
5. Store aliquots at 4°C for subsequent analyses with micro-CT, UV-visible spectroscopy analysis, and pH measurements.

### Micro-CT imaging

**Timing: 8 days total (For each sample at each time point: 14 min for lower-resolution analysis; at day 24: 64 min and 168 min for mid- and high-resolution analyses, respectively)**

This section describes the acquisition of micro-CT scans at multiple resolutions to monitor iodine uptake and visualize tissue microstructure. Consistent scanning and reconstruction parameters ensure accurate radiodensity quantification and structural analysis across time points.

6. Scan samples and the 1 ml iodine aliquots at each time point using the micro-CT scanner. The voxel resolution (25 or 30 μm) is selected based on the size of the sample and the field of view available on the scanner used.
7. At the final time point (day 24), acquire scan of selected regions of interest of the samples at both mid-resolution (8.5 μm of voxel size) and high-resolutions (2.5 μm of voxel size), to enable detailed structural analysis.

**CRITICAL:** Wrap the samples in parafilm to avoid tissue dehydration. Additionally, scan duration was kept short (14 min) to further minimize dehydration during imaging.

8. Reconstruct CT datasets using the filtered back-projection algorithm with the software provided by the system (X-Act v. 1.1, revision 22.11.1 2023-01-06, Rx Solutions).

**CRITICAL:** Perform the low-resolution analysis for the samples at each time point using the same micro-CT analysis settings and reconstruction parameters. This step is essential for accurately calculating the Hounsfield unit values of the menisci (as outlined in Data Processing and Analysis).

***Note:*** Beam-hardening correction was not applied during reconstruction, as no significant artifacts or grayscale distortions were observed under the imaging conditions used. The iodine concentration and the imaging setup used contributed to minimizing beam-hardening effects across all samples.

### Data processing and analysis

**Timing: 7 days**

This section describes the processing and quantitative analysis of micro-CT data to evaluate meniscal volume changes, iodine diffusion, and tissue radiodensity. Segmentation, radiodensity measurements, and region-specific analyses allow detailed assessment of iodine uptake dynamics over time.

9. Use Fiji and Avizo software to analyze the meniscal CT datasets.
  a. Use IsoData algorithm to segment the meniscal tissue.
    **CRITICAL:** Due to potential variability in grayscale values caused by iodine saturation or segmentation inaccuracies, a global thresholding method was not used. Instead, the IsoData thresholding algorithm is applied, enabling for adaptive segmentation based on the specific grayscale histogram of each dataset. This improves segmentation accuracy and consistency across samples. Manual verification of the segmented regions is recommended to ensure reliability.
  b. Normalize the segmented volumes to the original sample volume (day 0). To account for inter-sample variability, meniscal volumes were normalized by setting the original volume at day 0 as the maximum for each individual sample. Volume reduction during the staining period was then calculated relative to this baseline. This normalization approach minimizes variability between samples and enables direct comparison of results across different specimens.
  c. Calculate volumetric values at each staining time point. Sample volume was determined in Avizo by segmenting the meniscus using a threshold range from the IsoData value up to the maximum grayscale value (65535 for 16-bit images). The volume was then measured using the Volume Fraction tool within the Measure and Analyze module.
  d. To visualize the distribution of contrast agent in the meniscus: create 3D volume renderings using the Maximum Intensity Projection (MIP) technique and average 150 spatially consecutive radial meniscal slices for each time point.
  e. Convert the original CT datasets into an 8-bit format using the Convert Image Type module in Avizo.
    **CRITICAL:** Check that the input range is 0 – 65535 (16 bit image) and the output range is 0 – 255 (8 bit image), as this is important for the accurate calculation of radiodensity values.
  f. Measure the linear attenuation coefficient (μ) for each sample at specific staining times using the Label Analysis module in Avizo.
    ***Alternatives:*** The open-source software ImageJ can be used as an alternative imaging analysis tool.
  g. Calculate the Hounsfield Unit (HU) values to provide a quantitative standardize measure of the tissue radiodensity using the formula:
    (Equation 1) $$Hounsfield\;Unit=\frac{\left(\mu \text{tissue}-\mu \text{water}\right)}{\left(\mu \text{water}-\mu \text{air}\right)}\times 1000$$
    **CRITICAL:** To calculate the linear attenuation coefficient of water analyze 1 ml of water using micro-CT with the same settings and reconstruction parameters as those applied to the meniscal samples.
10. Use MATLAB to determine the contrast agent distribution.
  a. Define a region of interest extending from the outer periphery to the inner regions of the meniscus with radial width dimensions varying depending on the species and samples and a fixed height of 0.25 mm.
  b. Average the values from 100 CT slices taken from anatomical radial slices of the center of the meniscus.
    %% Section 1: Load subject folders and set processing parameters
    % Select the main folder containing the subjects
    mainFolder = uigetdir([], 'Select Main Folder'); (e.g., top-level folder with all samples)');
    % Retrieve subfolder names (excluding hidden/system folders)
    folderInfo = dir(mainFolder);
    subjectFolders = {folderInfo([folderInfo.isdir] & ∼startsWith({folderInfo.name}, '.')).name};
    % Create absolute paths for each sample folder
    imageFolders = fullfile(mainFolder, subjectFolders);
    %% Define processing parameters
    % Set the height (in slices) and radial width (in voxels) of the region of interest (ROI)
    % Adjust these values depending on tissue size, scanner resolution, or species
    commonHeight = 400; % Number of slices along the vertical axis of ROI (e.g., for ∼10 mm at 25 μm voxel size)
    commonWidth = 10; % Radial width of ROI in voxels (change depending on meniscus size)
    % Define file names corresponding to each staining time point
    % Make sure file names match the actual filenames in each folder
    fileNames = {'day0', 'day1', 'day4', 'day8', 'day12', 'day16', 'day20', 'day24'}; %correspond to timepoints
    timePointsLabels = [0, 1, 4, 8, 12, 16, 20, 24]; % Used for plotting and time-resolved analysis
    %% Set reference grayscale levels
    % These will be used to convert grayscale values to relative contrast values
    % GL_air can usually be set to 0 for air background
    GL_air = 0;
    % Ask the user to enter the grayscale value corresponding to water in the HU phantom
    % This is used to normalize grayscale values across samples/scanners
    GL_water = input('Enter the Grey Level of micro-CT HU phantom (corresponding to water): ');
  c. Convert the gray levels of the images to HU using the formula described above (Equation 1).
    %% Section 2: Compute normalized iodine concentration profiles for each subject
    % Initialize variable to store all concentration profiles (across subjects and time points)
    allConcentrationProfiles = [];
    % Loop through each subject folder
    for folderIdx = 1:length(imageFolders)
     imageFolder = imageFolders{folderIdx}; % Full path to the current subject folder
     concentrationProfiles = [];   % Will store profiles for all time points (columns)
     HU_max = -Inf;   % Initialize max HU for normalization
     % First pass: Compute maximum HU across all images
     for k = 1:length(fileNames)
      filePath = fullfile(imageFolder, [fileNames{k}, '.tif']);
      HU = computeHU(filePath, GL_air, GL_water); % Convert grayscale to HU
      HU_max = max(HU_max, max(HU(:)));   % Update global max HU
     end
    %% Compute HU for day 0 (baseline) and its mean
     filePath_day0 = fullfile(imageFolder, 'day0.tif');
     HU_day0 = computeHU(filePath_day0, GL_air, GL_water);
     HU_day0_mean = mean(HU_day0(:)); % Mean baseline signal for normalization
     %% Process each time point and compute relative concentration
     for k = 1:length(fileNames)
      filePath = fullfile(imageFolder, [fileNames{k}, '.tif']);
      HU = computeHU(filePath, GL_air, GL_water);
      % Normalize concentration relative to day 0 and maximum uptake
      if strcmp(fileNames{k}, 'day0')
      concentration = zeros(size(HU)); % No iodine uptake at baseline
      else
      % Normalize to percentage scale: 0% (day0) to 100% (max HU)
      concentration = ((HU - HU_day0_mean) / (HU_max - HU_day0_mean)) ∗ 100;
      end
      % Average over columns (2D slice): returns 1D radial profile
      avgConcentration = mean(concentration, 2);
      concentrationProfiles = [concentrationProfiles, avgConcentration];
     end
     %% Append results. Store 3D array: [rows × time points × subjects]
     allConcentrationProfiles = cat(3, allConcentrationProfiles, concentrationProfiles);
  d. Calculate the percentage of the contrast agent uptake (U%) using the formula:
    (Equation 2) $$U\%=\frac{\left(HU-HUday0\right)}{\left(HUmax-HUday0\right)}\times 100$$
    ***Note:*** HU is the Hounsfield Unit value of the current image, HU_day0_ is the mean HU value of the day 0 image (baseline without contrast agent), and HU_max_ is the maximum HU value observed across all images.
  e. Calculate the uptake along the width of the meniscus by averaging the HU values across columns for each image.
  f. Combine across all time points and samples the uptake profiles to calculate a mean uptake profile.
    %% Section 3: Compute and visualize mean concentration profiles across subjects
    % Average concentration profiles across all subjects (dimension 3 of the array)
    meanConcentrationProfiles = mean(allConcentrationProfiles, 3);
    % Create normalized depth axis (0 = outer edge, 1 = inner edge of the meniscus)
    depth_normalized = linspace(0, 1, size(meanConcentrationProfiles, 1));
    % Plot the results as a 2D heatmap
    figure;
    imagesc(depth_normalized, 1:length(fileNames), meanConcentrationProfiles');
    colormap(jet);
    colorbar;
    % Customize plot appearance
    xlabel('Normalized Depth');
    ylabel('Time Points (days)');
    title('Mean Concentration Profiles as a Function of Meniscus Depth');
    yticks(1:length(fileNames));
    yticklabels(timePointsLabels);   % Use defined time labels
    gca.XAxisLocation = 'top';    % Move x-axis to top
    colorbar.Label.String = 'Concentration (%)';
    colorbar.Limits = [0 100];    % Fix scale to allow comparison
    %% Save processed data
    % Save MATLAB .mat file for further processing
    save('mean_concentration_profiles.mat', 'meanConcentrationProfiles', 'depth_normalized');
    % Also save as CSV table for accessibility and external use
    T = array2table(meanConcentrationProfiles, 'VariableNames', string(timePointsLabels));
    writetable(T, 'mean_concentration_profiles.csv');
    %% Supporting Function: Convert grayscale to HU
    % This function linearly maps grayscale values to Hounsfield Units (HU)
    % based on the calibration between air (0) and water (entered by user)
    function HU = computeHU(filePath, GL_air, GL_water)
     img = imread(filePath);
     % Resize image to standard dimensions (height × width of ROI)
     % Adjust [400, 10] to match ROI dimensions used earlier in the script
     img = imresize(img, [400, 10]);
     % Apply HU conversion: 0 = air, 1000 = water
     HU = ((double(img) - GL_air) / (GL_water - GL_air)) ∗ 1000;
    End
    ***Note:*** To facilitate comparison the width of the menisci was normalized from 0 to 1.
11. Use Avizo software to calculate the radiodensity values of the staining solutions.
  a. Convert the original CT datasets into an 8-bit format using the Convert Image Type module in Avizo and selecting as output range 0–255.
  b. Measure the linear attenuation coefficient (μ) for each specific staining times using the Label Analysis module in Avizo.
  c. Calculate the Hounsfield Unit (HU) values to provide a quantitative standardize measure of the tissue radiodensity using the equation described above (Equation 1).
  d. Determine the percentage change in radiodensity by comparing the HU values from day 0 to day 1 of the staining solutions.

### UV-vis spectroscopy analysis

**Timing: 4 h**

This section describes the use of UV-visible spectroscopy to quantify iodine ion concentrations in the staining solutions at selected time points. The resulting data complement micro-CT measurements and allow assessment of iodine speciation and uptake dynamics during tissue staining.

12. Perform UV-visible spectroscopy to measure iodine ion concentrations in staining solutions.
  a. Dilute the staining solution by a factor of 500 to identify the ions present in solution.
  b. To measure I^-^ (peak at 228 nm) and I_3_^-^ (peaks at 288 and 351 nm) dilute the solutions 3000 and 500 times, respectively.
  c. Analyze the water- and PBS-based iodine solutions at days 0, 1, and 24 to investigate the uptake of iodine ions by meniscal tissue (Table 2).

***Note:*** Before samples analysis, record a solvent baseline measurement (water or PBS) for use as a blank sample.

***Note:*** UV-Vis spectroscopy was used to verify iodine ion speciation in solution and to support micro-CT findings related to diffusion behavior. Beyond its application to porcine and ovine meniscal tissue, this methodology can also be extended to menisci from other species and to other soft tissues, supporting the optimization of staining parameters for contrast-enhanced imaging tailored to specific needs. Future applications of the protocol could also expand the number of time points and directly correlate UV-Vis absorbance peaks with radiodensity measurements, providing deeper insight into the kinetics of iodine uptake.

**Table 2:** UV-vis spectroscopy parameters

Resolution and scan rate	Range of analysis	Identified peaks
1.5 nm and 300 nm/min	210–800 nm	228, 288, and 351 nm

### pH analysis

**Timing: 30 min**

This section describes the pH measurements of iodine staining solutions at selected time points. Monitoring pH changes helps assess solution stability and potential chemical changes during the staining process.

13. Perform pH analysis of the staining solutions.
  a. Measure pH values of both water- and PBS-based iodine solutions at days 0, 1, and 24 of the staining period.

## Expected outcomes

Application of this protocol allows researchers to quantify iodine uptake in meniscal tissues through both volumetric and radiodensity analyses. During the staining period, menisci typically exhibit progressive volumetric shrinkage accompanied by an increase in radiodensity, reflecting iodine penetration into the tissue matrix. 3D micro-CT imaging enables visualization of iodine diffusion patterns across the meniscal structure, while UV-Vis spectroscopy allows identification of iodine ion species present in the staining solutions and their dynamic changes over time. Collectively, these outcomes provide detailed information on iodine diffusion kinetics and tissue interaction, supporting the optimization of contrast-enhanced imaging protocols for soft tissues.

## Limitations

The staining process for meniscal tissue requires up to 24 days, which may not be compatible with all experimental timelines. Despite the extended staining duration, there remains a risk of incomplete or non-uniform contrast agent penetration, particularly in larger samples. Differences in meniscal tissue properties between sheep and pigs can also affect iodine uptake and diffusion patterns, potentially complicating cross-species comparisons. Proper fixation in formaldehyde is critical, as incomplete fixation may compromise tissue quality and reproducibility. Finally, tissue dehydration during imaging, even when samples are wrapped in parafilm, can alter tissue volume and radiodensity, thereby impacting image quality and data accuracy.

## Troubleshooting

### Problem 1

Low radiodensity contrast (related to step 6).

Low radiodensity contrast can occur when the sample does not exhibit enough variation in X-ray absorption, making it difficult to distinguish different structures in imaging.

### Potential solution

• Increase staining duration: Extending the staining time allows for better penetration of contrast agents.
• Increase iodine concentration: Raising the concentration of iodine in the stain can lead to greater contrast, helping to distinguish finer details in the sample.

### Problem 2

Noise and Artifacts in X-ray Imaging (related to step 6).

Noise and artifacts are common issues in X-ray micro-CT imaging that can degrade image quality and hinder accurate analysis. These issues can result in inaccurate representations of the sample, making it challenging to interpret fine details or perform precise measurements.

### Potential solution

• Optimize scan settings: Adjusting the voltage, current, and scan resolution can minimize noise while maintaining image quality.
• Motion correction: Ensure the sample is properly stabilized during scanning to prevent motion artifacts.
• Beam hardening correction: Apply beam hardening correction algorithms to reduce.
• Filtering and denoising: After imaging, applying post-processing techniques like spatial filtering or denoising algorithms can help reduce noise and improve the clarity of the final images.

## Resource availability

### Lead contact

Further information and requests for resources and reagents should be directed to and will be fulfilled by the lead contact, Annapaola Parrilli (annapaola.parrilli@empa.ch).

### Technical contact

Technical questions on executing this protocol should be directed to and will be answered by the technical contact, Federica Orellana (federica.orellana97@gmail.com).

### Materials availability

This study did not generate new unique reagents.

### Data and code availability

Upon appropriate agreement, the generated datasets can be requested from the lead contact.

## Acknowledgments

This work was supported by the 10.13039/501100001711Swiss National Science Foundation (grant no. 197928).

## Author contributions

This protocol was developed and optimized by F.O. and A.P. The detailed procedure was written by F.O. and edited by A.P. Resources and funding were secured by A.P.

## Declaration of interests

The authors declare no competing interests.
